# A Simple Target Will Save the Day and the Kidney: This Is How We Perform Our Endovascular Fenestrated Graft Procedure

**DOI:** 10.7759/cureus.14641

**Published:** 2021-04-23

**Authors:** Matthew Montanarella, Taylor S Harmon, Carissa Concepcion, John Pirris, Jerry Matteo

**Affiliations:** 1 Radiology, University of Florida College of Medicine, Jacksonville, USA; 2 Surgery, University of Florida College of Medicine, Jacksonville, USA; 3 Cardiothoracic Surgery, University of Florida College of Medicine, Jacksonville, USA

**Keywords:** abdominal aortic aneurysm, fenestrations, endovascular aortic repair, renal artery target, renal artery occlusion, renal artery preservation

## Abstract

With the advent of state-of-the-art imaging modalities, increasing population age, and advanced preventive medical treatments, medical device design attempts to keep up with procedural demand. An abdominal aortic aneurysm (AAA) is a recognized, potentially fatal disease process where strides have been made in screening, detection, and treatment since its discovery. With the introduction of percutaneous endograft procedures in 1991, open surgical treatment is nearly a lost art. Endovascular aortic repair is now the gold standard. However, short landing zone necks, hostile angulation, and markedly dilated seal zones present challenges for one size fits all endovascular aortic devices. Suprarenal and juxtarenal fenestrated aortic grafts are the most advanced individually customized grafts invented to date. Subsequently, proper placement of these complex devices still presents challenges. We present a method for preoperative renal stent placement for target purposes. This article includes a pictorial guide and describes the tips and pitfalls for easy proper AAA exclusion with a fenestrated aortic graft. We were successful in the deployment of the fenestrated graft device and the exclusion of an aortic aneurysm while preserving the patency of the renal arteries. The patient had no postoperative complications. During 18-month postoperative surveillance, imaging demonstrated proper graft positioning without evidence of an endoleak. In fenestrated endovascular aortic repair, preoperative renal stenting is paramount for targeting purposes. This allows for the precise and timely deployment of the renal limbs through the fenestrations while minimizing the risk of postoperative complications, including renal artery occlusion.

## Introduction

Abdominal aortic aneurysms (AAAs) are defined as a segment of the aorta near the renal arteries with a diameter 1.5 times larger than normal or a diameter greater than 3.0 cm [1-2]. In the United States, AAA is the 14th leading cause of death and the 10th leading cause of death in older men [1,3]. An aneurysm occurs between the renal arteries and aortic bifurcation in 80% of cases [1]. Risk factors for AAA include smoking, age, sex, and family history, among others. It was discovered that smoking was the greatest risk factor for aneurysm formation with a graded increased association relative to the length of time smoking [4]. Recommendations by the United States Preventative Services Task Force (USPSTF) consist of one-time ultrasound in men ages 65-75 who have ever smoked to screen for AAA [5]. Most AAAs go unnoticed until they rupture, resulting in a mortality rate of around 60-80% [6-7].

The advent of endovascular repair for AAA materialized in 1991 by Parodi et al. [8]. Since the introduction of this technique, open surgical repair has nearly vanished as a primary treatment option. Following the publication of this landmark technique, numerous large multicenter clinical trials demonstrated the safety and efficacy of endovascular aortic repair (EVAR) [9-10]. In 1999, the Food and Drug Administration (FDA) approved five endovascular graft devices for the clinical use of AAA repair [11]. Along with advances in imaging technology came an increased ability to determine patient-specific anatomy and aneurysmal variation. This technological advancement led to the development of more complex, branched, bifurcated, and fenestrated grafts. Endovascular aortic repairs present some risk of post-procedure complications, including infection, endoleak, an embolic phenomenon with end-organ ischemia, graft migration, and renal artery occlusion [12].

The first fenestrated aortic graft was deployed to treat AAA in 1999 by Browne [13]. The juxtarenal zone is a critical area that can determine the success or failure of graft placement and the ultimate repair of AAA. Standard non-fenestrated devices used to repair short neck aneurysms have led to the increased incidence of proximal type 1 endoleak [14]. In addition to hostile angulation and markedly dilated seal zones, these short landing zone necks present challenges for a universal endovascular aortic device. To date, suprarenal fenestrated aortic grafts are the most advanced individually customized devices. These progressive devices still offer their own set of deployment challenges due to the possible hostile anatomy mentioned above. Even with meticulous preprocedural graft planning, intraoperative graft deployment may be less than satisfactory and require repositioning. We present a method for preoperative renal stent placement for radiographic target purposes. This article describes the tips and pitfalls for easy proper AAA exclusion with a fenestrated aortic graft.

## Technical report

Years of refining technical success have led from open surgical repair to various FDA-approved endovascular aortic repair methods. Presently, fenestrated endovascular aortic repair (FEVAR) stents are utilized to exclude AAA. Though the functionality of these devices has improved from older technology, FEVAR is not without caveats. FEVAR stents are successful for AAA exclusion, although there is an alarming high post-procedural renal devascularization rate. This is the result of intraprocedural failure to access the renal arteries from manufactured pre-cut fenestrations. We propose preprocedural stenting for all FEVAR cases to ensure renal artery preservation.

FEVAR stents are made-to-order devices that are custom assembled and tailored to specific patient anatomy. The patient's abdominal aorta, visceral segment, aortic bifurcation, and common iliac arteries are reconstructed using computed tomographic (CT) rendering software. Once a digitized model is completed, the manufacturer assembles the FEVAR stent. The device is sent to the ordering institution and deployed. Though the manufacturer software precisely calculates the renal artery ostia on a two-dimensional plane, it fails to account for the in-vivo three-dimensional tortuosity and angulation of the aorta, tortuosity of the iliac arteries, and spatial planes that the renal arteries may originate. These factors complicate intraoperative deployment. The operator may spend hours trying to access the native renal artery ostia blindly either above or below the pre-cut fenestration. The worst-feared yet common outcome is renal artery exclusion.

**Figure 1:**
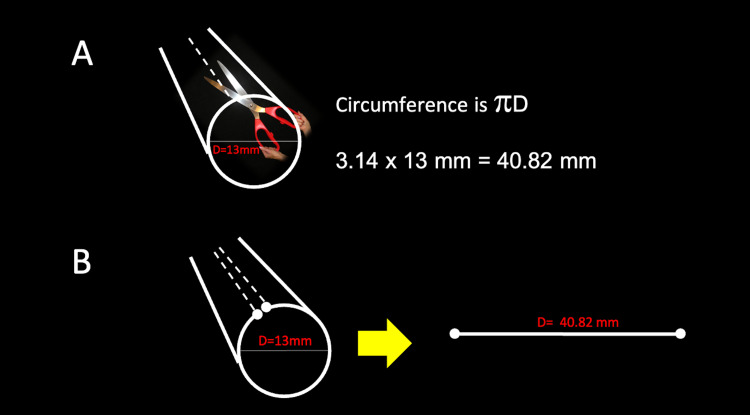
A schematic demonstrates that when using the diameter of an endovascular stent, the circumference is calculated (A) and equal to the stent’s length if it were cut open and displayed in a straight line (B). D: diameter; mm: millimeters

An example of how the manufacturer's CT-rendering software calculates pre-cut fenestrations into a custom FEVAR stent is demonstrated by observing the two-dimensional diameter of the native aorta in a short axis and calculating the diameter (Figure 1).

**Figure 2:**
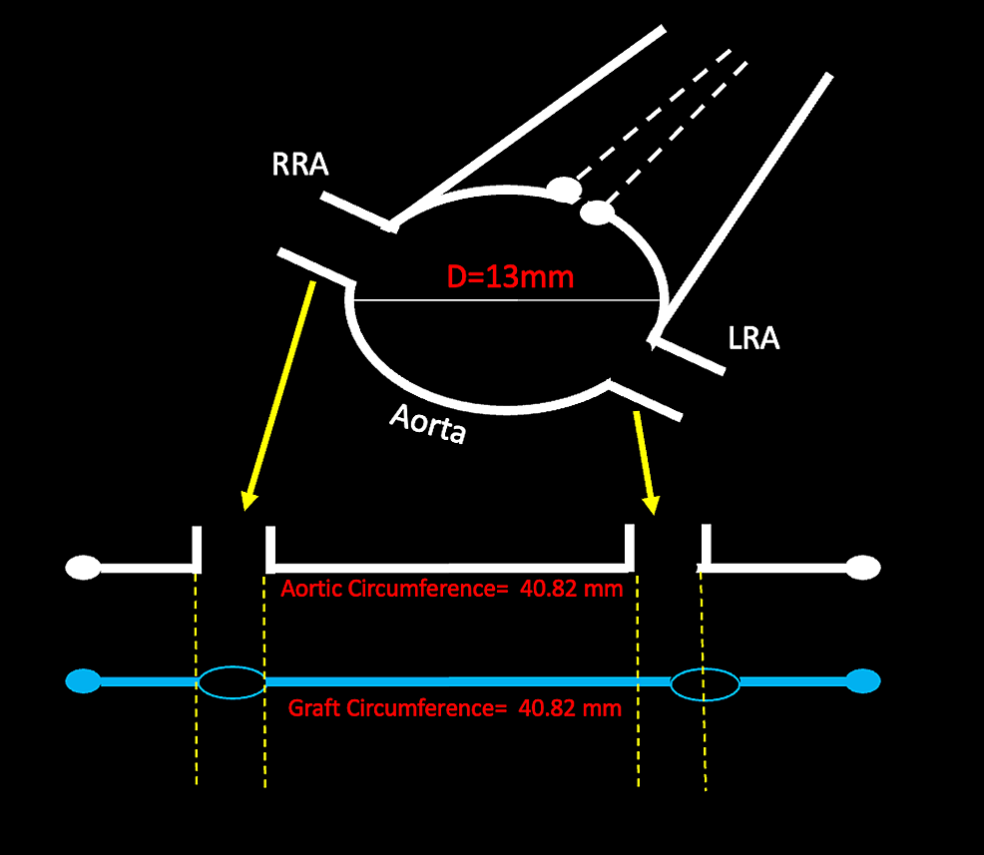
A schematic demonstrates how the manufacturer's CT-rendering software places two pre-cut fenestrations in the fenestrated endovascular aortic repair stent to reflect the renal artery ostia (yellow arrows). Although the two-dimensional native aortic circumference is the same as the graft circumference, the fenestrations may not accurately align (dashed yellow lines). RRA: right renal artery; LRA: left renal artery; D: diameter; mm: millimeters

The software selects two regions within the circumference and marks these areas for targeted fenestration. The software will then calculate a vessel circumference using the diameter, for example, 13 mm, or 22 mm, or 30 mm, which would correspond to 40.82 mm, 69.12 mm, and 94.25 mm, respectively. The software will apply two pre-cut fenestrations within the stent material to allow blood to flow into the opposing ostia (Figure 2).

**Figure 3:**
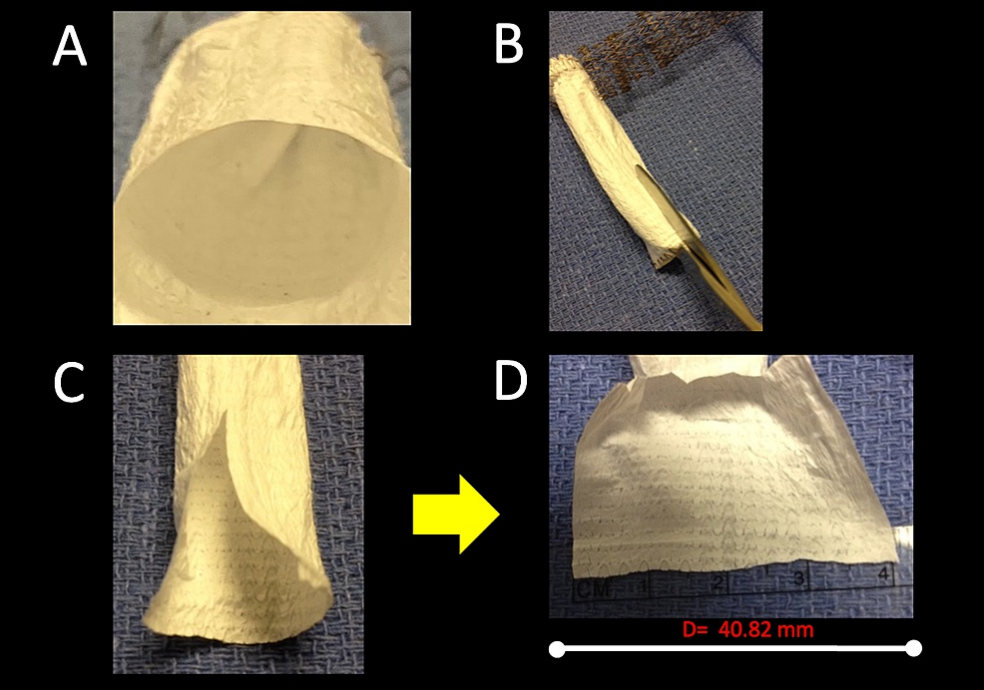
An in-vitro demonstration of cutting a 13-millimeter stent (A, B) in the long axis will display the stent circumference in a measurable straight line (C, D). In Figure 3D, a ruler shows the stent's circumference is 40.82 millimeters when displayed in two dimensions. D: diameter; mm: millimeters

For a visual demonstration, a 13 mm stent is cut, and the circumference is displayed as the linear distance to show how the manufacturer's CT-rendering software calculates an ideally fitted FEVAR stent for each unique patient (Figure 3).

**Figure 4:**
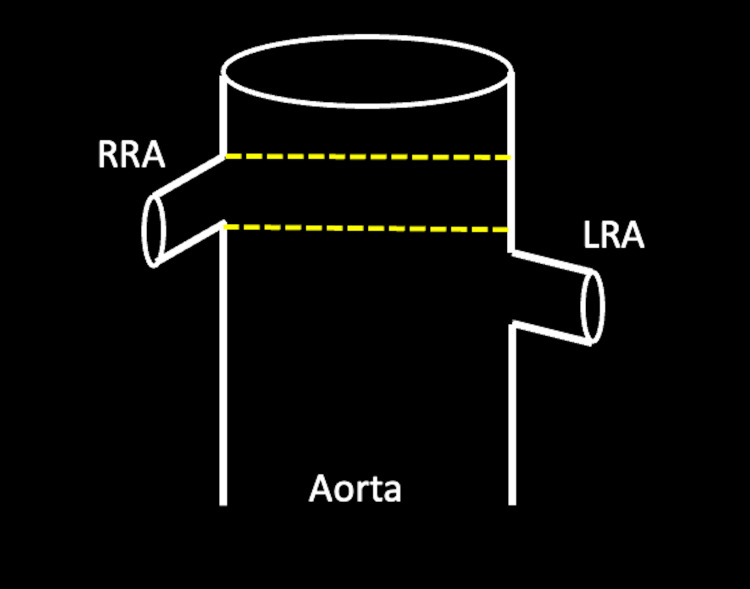
A schematic demonstrates that computed tomography-rendering software will not consider factors inherent to the patient's anatomy, even with an accurately fitting fenestrated endovascular aortic repair stent. This results in inadvertently misaligned pre-cut fenestrations (dashed lines). RRA: right renal artery; LRA: left renal artery

While the software aligns the pre-cut fenestrations in the two-dimensional plane based on the circumference of the native aorta, it fails to appreciate the dynamic elasticity, tortuosity, and angulation inherent to the aorta. A simple example demonstrates that even though the fenestration aligns with the left renal artery, the ostia is lower than expected (Figure 4).

**Figure 5:**
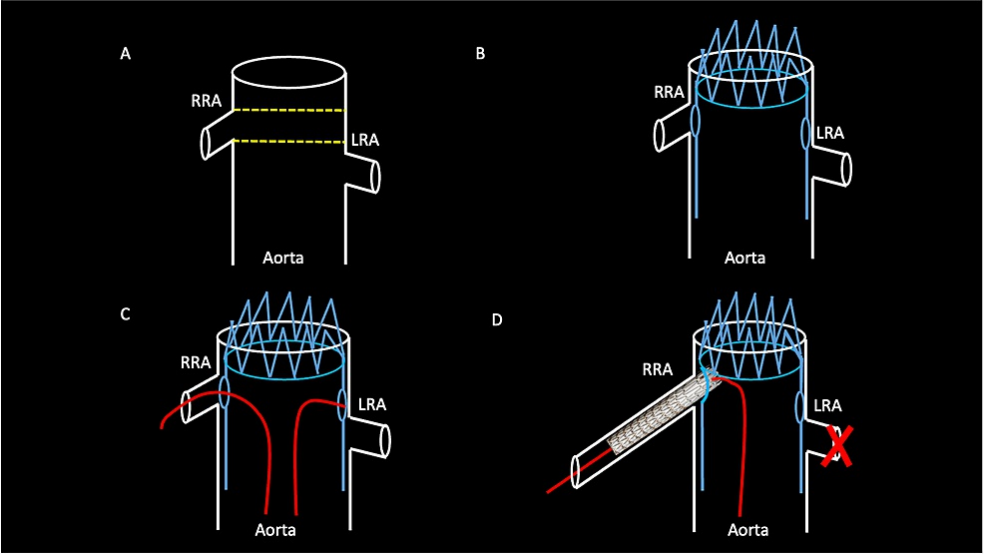
A schematic shows the consequences of the intraoperative fenestrated endovascular aortic repair without preprocedural renal stenting. Redemonstrated from Figure 4 is the malalignment of the fenestrated endovascular aortic repair stent and the patient’s native renal artery ostia. Dashed lines show the inadvertently misaligned pre-cut fenestrations (A). When the fenestrated endovascular aortic repair stent is placed, the pre-cut fenestrations may align with one renal artery ostium but will not account for the other, in this case, the left renal artery (B). When cannulation of the renal arteries is performed with a guidewire, one renal artery may be easily vascularized, but it is laborious or impossible to cannulate the other (C). The right renal artery and native aorta are bridged, but the left renal artery cannot be accessed, resulting in devascularization (D). RRA: right renal artery; LRA: left renal artery

These calculation discrepancies lead to intraoperative procedural mismatch. It can also lead to a devastating consequence of renal artery exclusion if an operator cannot access the mismatched fenestration (Figure 5).

**Figure 6:**
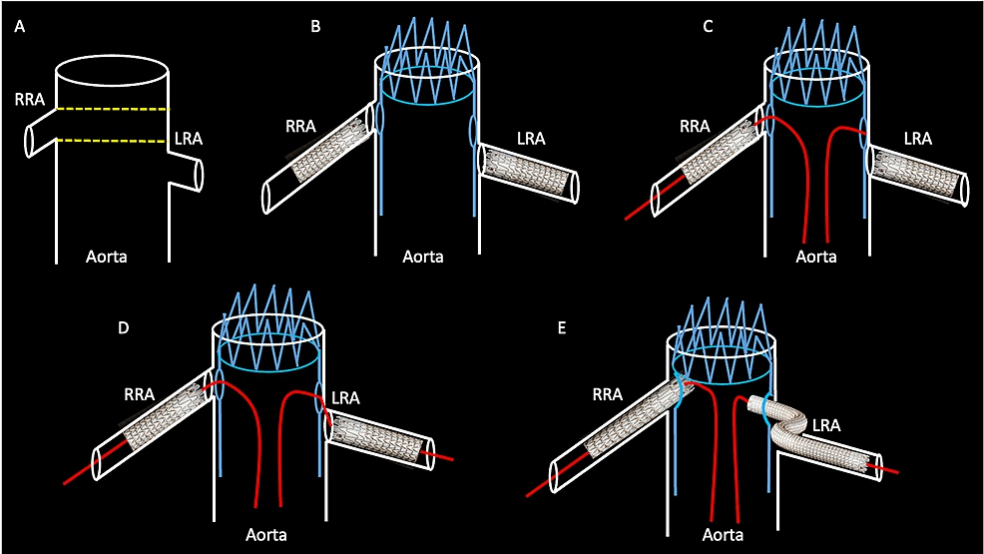
A schematic shows the benefit of preprocedural renal stenting before the placement of a fenestrated endovascular repair stent Redemonstrated from Figure 4 is the malalignment of the fenestrated endovascular aortic repair stent and the patient’s native renal artery ostia. Dashed lines show the inadvertently misaligned pre-cut fenestrations (A). Preprocedural renal stents are placed in the bilateral renal arteries to allow easier ostial bridging in the subsequent fenestrated endovascular aortic repair deployment (B). During deployment of the fenestrated endovascular aortic repair stent, one of the renal arteries is easily cannulated by a guidewire, in this case, the right renal artery (C). The radiopaque markers on the preprocedural renal stents are visible on angiography, allowing for easier targeted cannulation of the contralateral side (D). Once the abdominal aortic aneurysm is excluded, the bridging renal artery stents are deployed, allowing vascularization of the bilateral kidneys (E). RRA: right renal artery; LRA: left renal artery

The solution for this manufacturer discrepancy is simple and consists of preprocedural renal artery stenting. This method ensures targeted landing zones are accessible for the operator when the FEVAR stent is placed, allowing procedural efficiency and radiation reduction to the patient. Once the FEVAR stent is deployed, each subsequent renal artery is cannulated, and bridging stents seal the aortic lumen (Figure 6).

The following case demonstrates preprocedural renal stenting and subsequent FEVAR of a patient with a large infrarenal AAA, which successfully preserved the renal arteries in addition to AAA exclusion.

A 60-year-old male with an extensive smoking history presented with a large juxtarenal AAA, not amenable to standard endovascular aortic repair. We determined that the patient was a candidate for FEVAR with preprocedural renal artery stenting. The right common femoral artery was accessed using a micropuncture needle and sheath. A hydrophilic wire was advanced into the thoracic aorta, and the micropuncture sheath was exchanged for a 7-French vascular sheath over the wire. Selective catheterization of the left renal artery was performed, confirming catheter placement in the left main renal artery.

**Figure 7:**
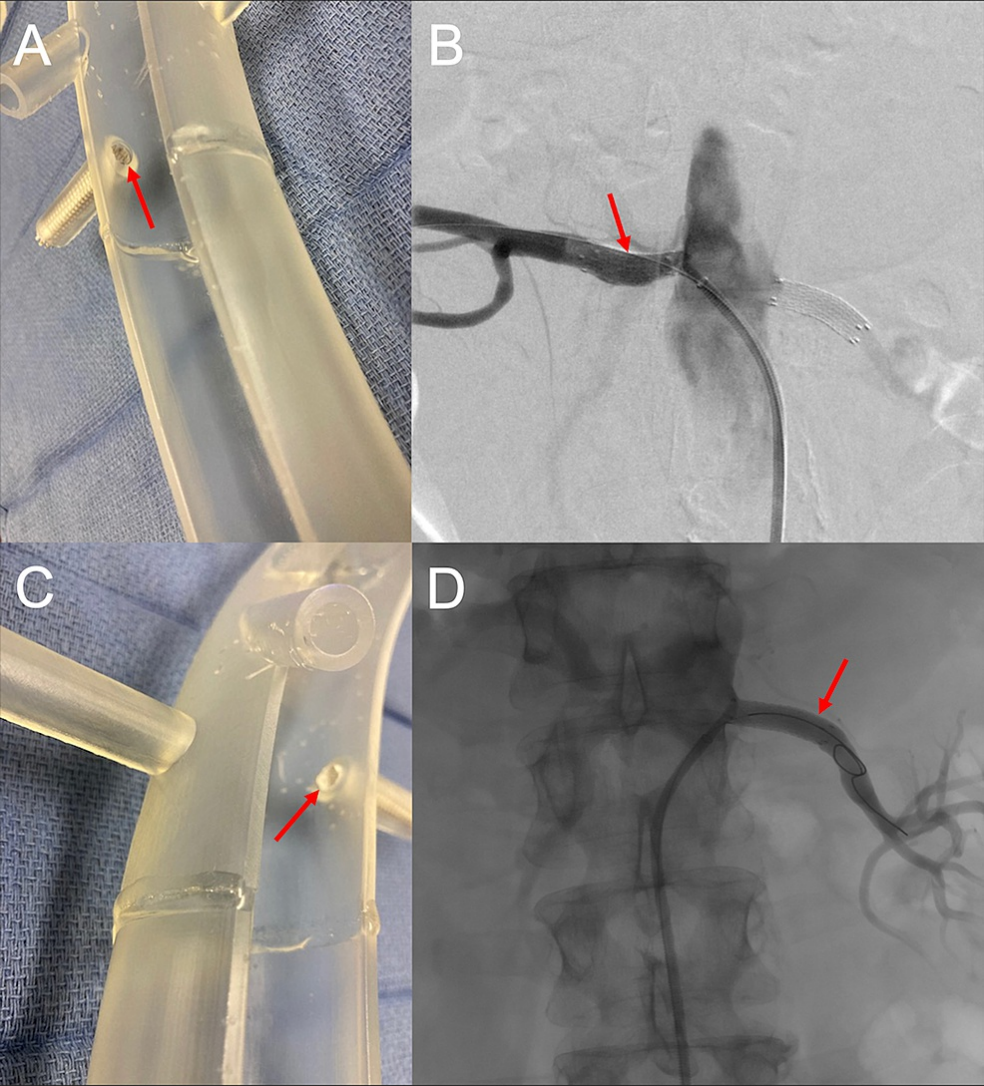
A three-dimensional printed acrylic model and intraprocedural angiogram demonstrate the importance of deploying the covered stents flush to the native aorta. This allows for the accurate subsequent deployment of the fenestrated endovascular aortic repair stent into the native aorta. The three-dimensional printed acrylic model renal artery ostium (A) shows the flush deployment of a covered renal artery stent (red arrow). The corresponding intraprocedural angiogram shows the covered renal stent within the right renal artery (red arrow) and vascularization of the right distal renal artery (B). The three-dimensional printed acrylic model renal artery ostium (C) shows the flush deployment of a covered renal artery stent (red arrow). The corresponding intraprocedural angiogram shows the covered renal stent within the left renal artery (red arrow) and vascularization of the left distal renal artery (D).

A 7 mm x 2.5 cm Viabahn® (W.L.Gore, Newark, DE) covered stent was successfully deployed across the left renal ostium. The stent was intentionally deployed flush with the aorta so that subsequent FEVAR would not compromise the renal stent. Following this, an 8 mm x 2.5 cm Viabahn covered stent was successfully deployed across the right renal artery ostium. As with the contralateral side, the stent was intentionally deployed flush with the aorta. A follow-up angiogram demonstrated excellent results without stent extension into the aortic lumen (Figure 7).

Approximately one month after ordering the custom-made FEVAR stent, the patient was scheduled for an outpatient procedure. The patient was placed on the angiography table under general endotracheal anesthesia. The right common femoral artery was then accessed using a micropuncture system. After introducing a stiff guidewire into the abdominal aorta, a 4-French micropuncture catheter was upgraded to a 20-French sheath.

**Figure 8:**
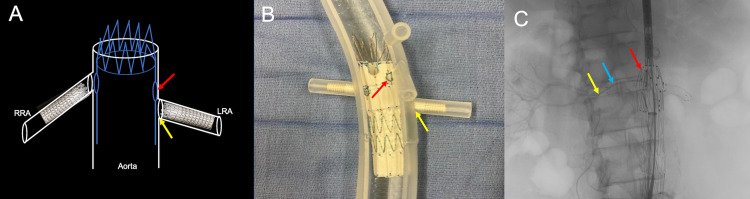
Corresponding schematic (A), three-dimensional printed acrylic model (B), and intraprocedural angiogram (C) demonstrate the deployment of a fenestrated endovascular repair stent into the native aorta. The red arrow shows the fenestrated endovascular repair stent deployment. The preprocedural renal artery stent helps the operator position the fenestrated endovascular repair stent (blue arrow). The yellow arrow shows the guidewire within the right renal artery. RRA: right renal artery; LRA: left renal artery

The left common femoral artery was accessed with a micropuncture system. Under fluoroscopic guidance and over a guidewire, the micropuncture sheath was exchanged for an 18-French sheath. A 5-French flush catheter with radiopaque markers was introduced into the abdominal aorta. Through the right common femoral artery groin sheath and over a guidewire, the main body of the custom-made Zenith® (Cook Medical, Bloomington, IN) fenestrated AAA endovascular graft was advanced into the infrarenal aorta. The FEVAR stent was deployed with the proximal aspect above the renal arteries at the superior mesenteric artery level. The bilateral preprocedural stents were used to guide the placement of the primary device (Figure 8).

**Figure 9:**
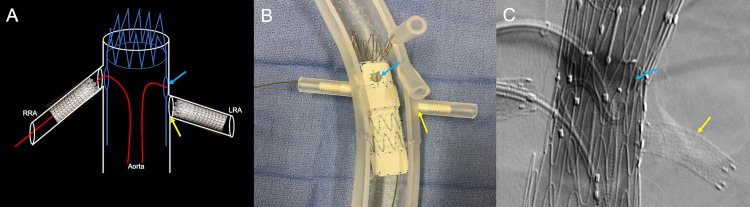
Corresponding schematic (A), three-dimensional printed acrylic model (B), and intraprocedural angiogram (C) demonstrate the misalignment of the fenestration with the left renal ostium. Figure 9A demonstrates the failure of a wire (red line) to negotiate from the fenestration (blue arrow) through the left renal ostium (yellow arrow). Figure 9B demonstrates an acrylic model with the fenestrated graft in place and a guidewire successfully cannulating the right renal artery. In this model, the graft fenestration (blue arrow) is markedly misaligned with the left renal artery ostium (yellow arrow). Figure 9C demonstrates a lateral projection of procedural aortic angiogram in an attempt to visualize the left renal artery. The aortic graft is widely patient (blue arrow); however, the left renal artery and preprocedural stent are occluded by the graft wall and non-opacified (yellow arrow). RRA: right renal artery; LRA: left renal artery

**Figure 10:**
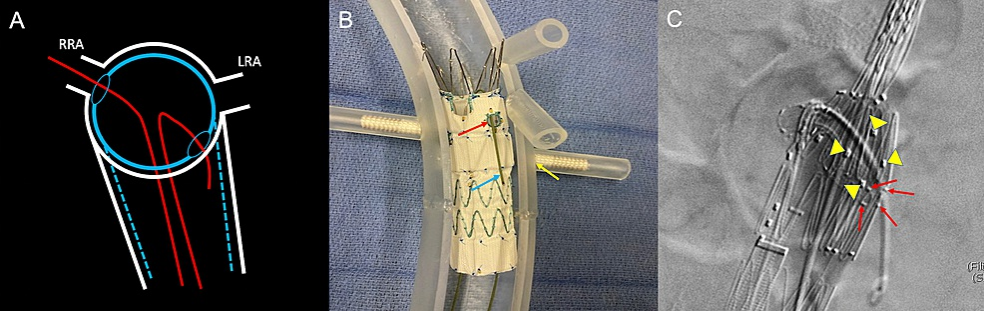
Corresponding schematic (A), three-dimensional printed acrylic model (B), and intraprocedural angiogram (C) demonstrate the difficulty of cannulating the opposing renal artery during the deployment of a fenestrated endovascular aortic repair stent due to the described manufacturer's CT-rendering software limitations. In Figure 10B, the guidewire (blue arrow) is traversing the pre-cut fenestration (red arrow), showing an in-vitro view of how the operator must access the renal artery (yellow arrow) through the pre-cut fenestration. Figure 10C shows how the radiopaque markers on the preprocedural renal stent (yellow arrowheads) guide the cannulation of the renal artery. The red arrows show the pre-cut fenestration on the fenestrated endovascular aortic repair stent and the discontinuity between the stent and renal artery ostium. RRA: right renal artery; LRA: left renal artery

The initial catheterization of the right renal artery demonstrated the wire's passage beyond the lumen of the existing right renal artery stent. Due to the manufacturer mentioned above - CT rendering software limitations - the left renal artery ostium's inferior positioning was not easily accessible. However, the radiopaque markers from the preprocedural renal artery stents within the left renal artery guided the wire positioning during intraprocedural angiography (Figure 9 and Figure 10).

**Figure 11:**
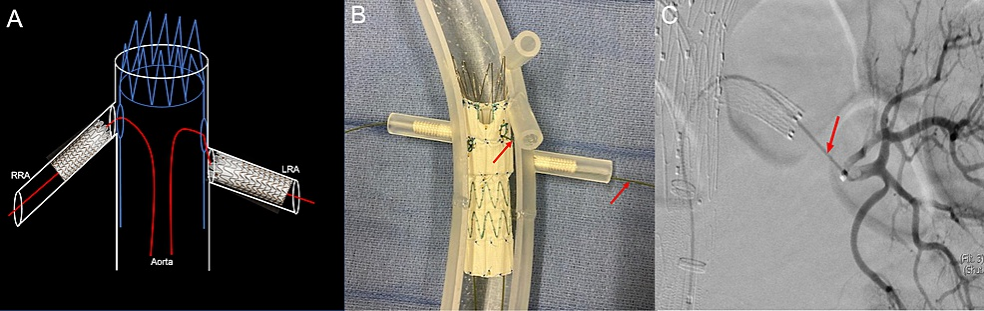
The corresponding schematic (A), three-dimensional printed acrylic model (B), and intraprocedural angiogram (C) demonstrate the cannulation of the left renal artery (red arrows). In Figure 11C, a microcatheter (red arrow) was used to cannulate the graft fenestration and renal artery ostia. Complete vascularization of the left renal artery is shown. RRA: right renal artery; LRA: left renal artery

Once the left renal artery was cannulated, a subsequent angiogram demonstrated the appropriate vascularization of the kidney (Figure 11).

**Figure 12:**
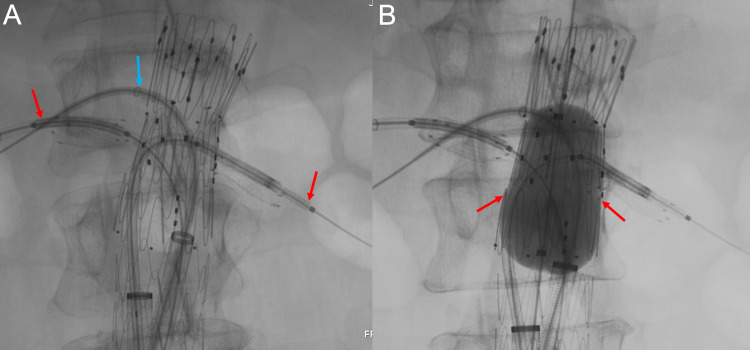
Once the bilateral renal arteries are cannulated (A), two bridging stents are advanced through the preexisting renal artery stents (red arrows). The blue arrow shows the cannulation of the superior mesenteric artery. Before deploying the bridging stents, the abdominal aortic aneurysm is excluded (B) with an intra-aortic balloon (red arrows).

An 8 mm x 29 mm VBX® (W.L.Gore, Newark, DE) stent was advanced into the left renal artery. A 6 mm x 39 mm VBX stent was advanced into the right renal artery, both through the previously existing renal artery Viabahn stents, before deploying the VBX stents. The FEVAR stent was dilated with a balloon to exclude the AAA completely (Figure 12).

**Figure 13:**
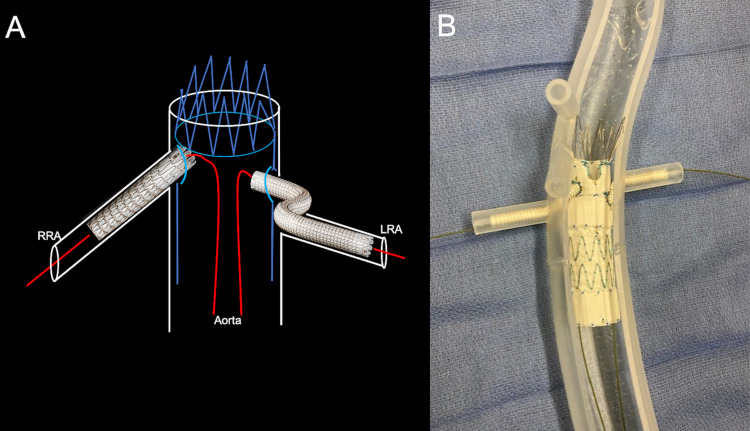
Corresponding schematic (A) and three-dimensional printed acrylic model (B) demonstrate the deployment of the bilateral bridging renal artery stents after balloon-dilated exclusion of the abdominal aortic aneurysm. RRA: right renal artery; LRA: left renal artery

The bilateral VBX stents were then deployed within the preprocedural renal Viabahn stents, bridging the renal arteries and inflow from the native aorta (Figure 13).

**Figure 14:**
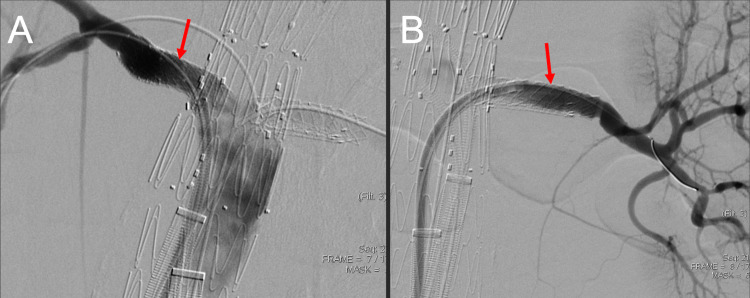
The right renal artery (A) and left renal artery (B) angiograms demonstrate contrast patency of the renal arteries after bridging of the renal arteries and native aorta. The red arrows show the bridging stents within the bilaterally placed preprocedural renal artery stents.

Bilateral renal artery angiograms were performed to demonstrate the vascularization of the bilateral kidneys (Figure 14).

**Figure 15:**
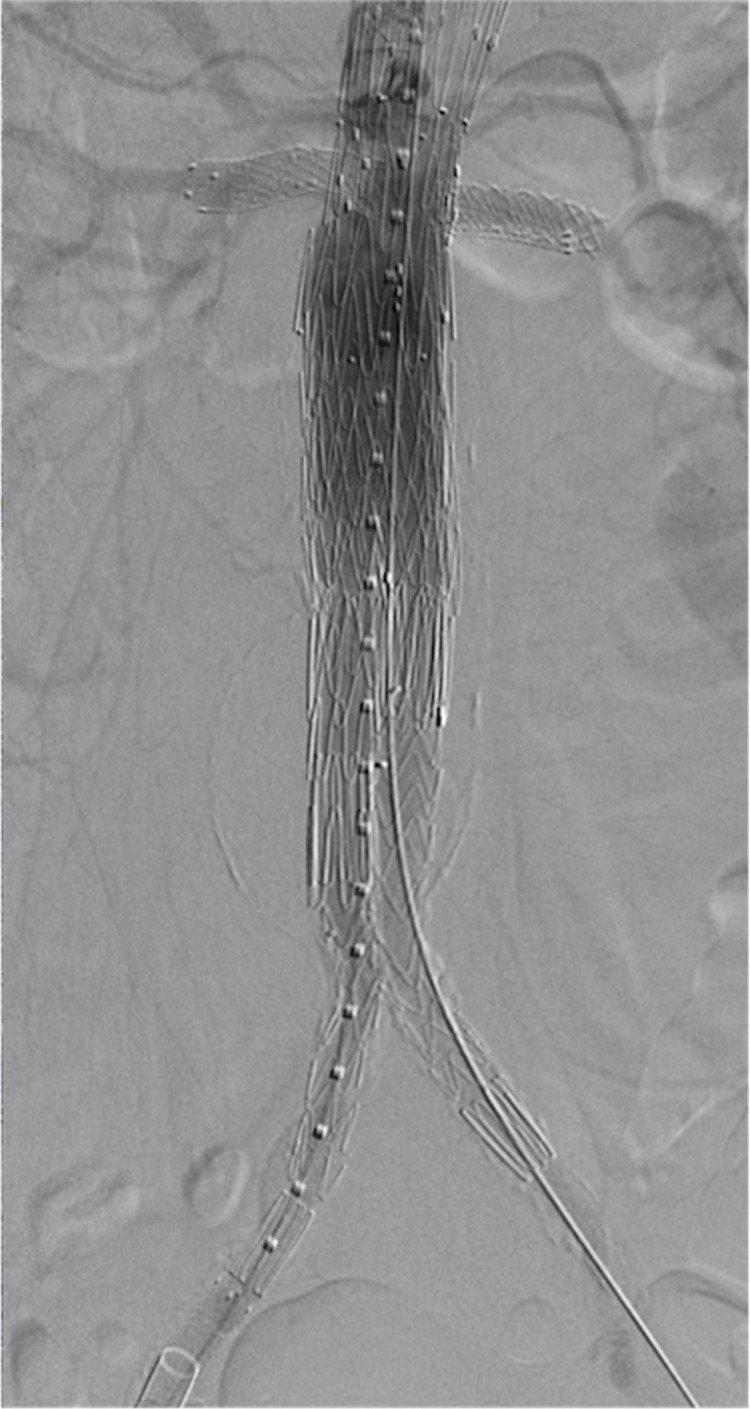
A final aortogram demonstrates successful contrast patency throughout the bilateral renal arteries and bilateral common iliac limbs. Additionally, the abdominal aortic aneurysm was excluded entirely.

The subsequent steps for successful FEVAR stent deployment were conducted without incident. Post-deployment angiography through the bilateral groin sheaths was then performed. This demonstrates the patency of the graft lumen and appropriate positioning (Figure 15).

Sterile dressings were applied after all the wires and catheters were removed, and hemostasis was achieved. In the postoperative period, the patient was extubated and admitted to the intensive care unit for close monitoring and overnight observation.

**Figure 16:**
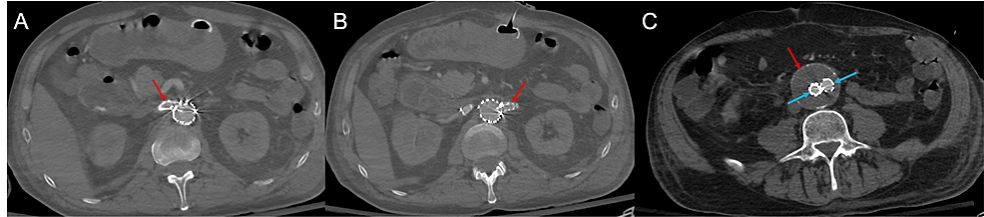
Surveillance computed tomography angiogram shows stent patency throughout various levels in the axial orientation. The right renal artery (A) and left renal artery (B) continue to demonstrate contrast patency (red arrows). A lower level in the axial orientation (C) shows the continued exclusion of the abdominal aortic aneurysm (red arrow) at the level of the common iliac arteries (blue arrows).

**Figure 17:**
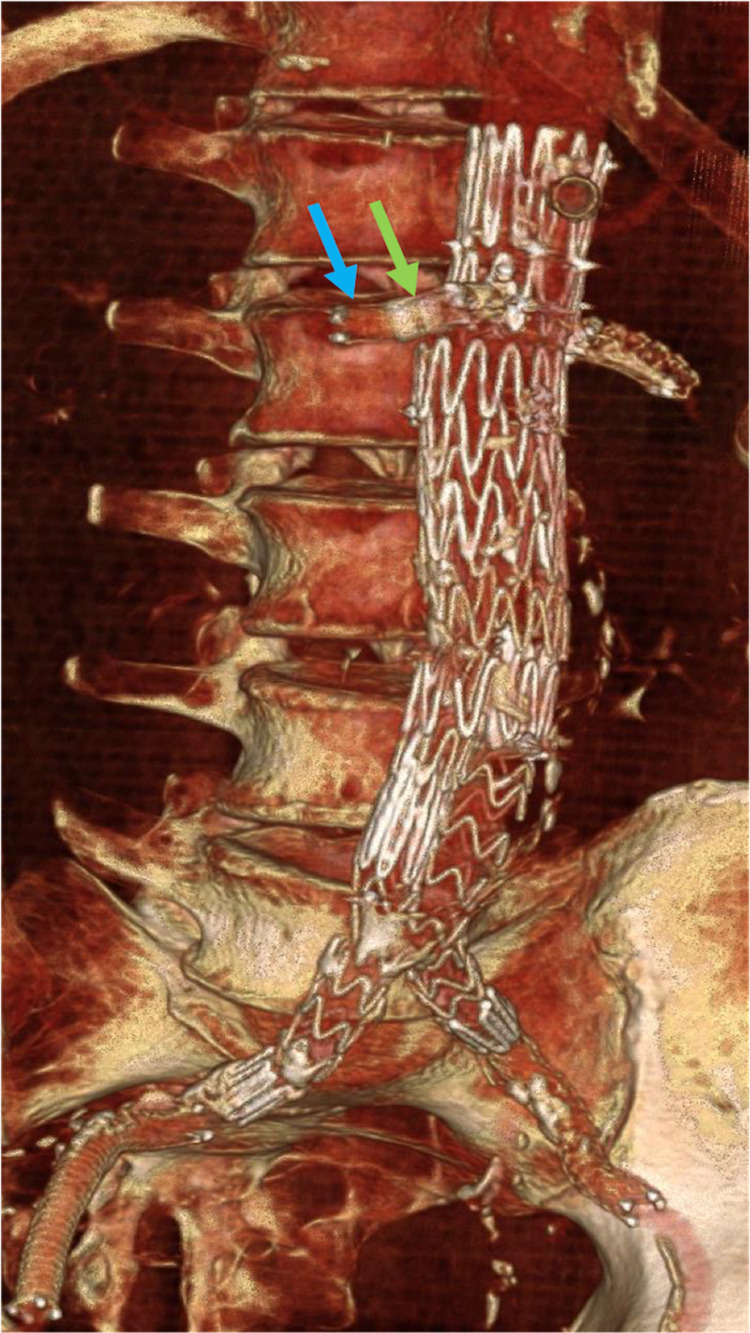
Surveillance computed tomography-based volume-rendered projection shows the patency of the fenestrated endovascular aortic repair stent. The bilateral renal arteries also remain patent. The bridging renal artery stents (blue arrow) remain patent within the preprocedural renal artery stents (green arrow).

Throughout the 18-month, post-procedural surveillance period, satisfactory positioning of the FEVAR stent components without evidence of type I or III endoleaks has been demonstrated. Patency has been identified throughout the bilateral renal artery bridging stents, native aorta, and bilateral common iliac arteries. The entirety of the infrarenal AAA has continued to be excluded (Figure 16 and Figure 17).

## Discussion

Compared to AAA's open surgical repair, an endovascular approach is less invasive, less costly, has higher technical success, and decreased 30-day all-cause mortality [1]. Unintentional deployment of a fenestrated stent graft and occlusion of one or both renal arteries could result in catastrophic renal failure and long-term dialysis [12]. For incorrect placement cases or fenestrated mismatch, attempts can be made to utilize a “pull-down” maneuver with an angioplasty balloon placed inside the existing graft to move it caudally into the correct position. A study of 72 patients receiving fenestrated endovascular aortic repair demonstrated a complication rate of 24% [15]. These complications are renal artery occlusion, renal artery stenosis greater than 60%, or a decrease in GFR following the procedure requiring hemodialysis [15]. A complication rate of 24% is unacceptable and a method to combat this alarming percentage is imperative.

Renal stenting before fenestrated graft placement provides a target for assisting in proper device deployment and avoiding future graft adjustments altogether by increasing successful positioning on the first attempt. Before fenestrated grafts were available in the United States, treating patients with short infrarenal necks required intraoperative “periscope” or “snorkel” methods of renal stenting to preserve renal perfusion [16]. Previous methods for choosing proper size stents were the only endovascular options [16]. FEVAR negates the need for a renal snorkel. Short infrarenal necks, hostile renal artery angulation, and dilated proximal seal zones create difficulty intraoperatively during fenestrated graft placement and increase the likelihood for renal artery occlusion. Without careful preoperative planning and a precise procedural technique, the case may have to be converted to an open surgical approach, which is considered a higher risk than a primary open procedure [17].

Fenestrated endovascular aortic repair utilizes a large amount of radiation exposure and contrast. A study of 72 patients receiving three or four-vessel FEVAR, radiation dose, and contrast usage were, on average, 5400±2225 mGy*cm2 and 90±25ml, respectively [18]. Two-vessel FEVAR had an average fluoroscopy time of 63 minutes, and three or four-vessel procedures had a fluoroscopy time of 89 minutes [18]. Compared to traditional endovascular aortic repair (EVAR), cases had three to four times less fluoroscopy time with an average of 21.8 minutes [19]. The amount of radiation exposure would only increase in a difficult FEVAR case with inaccessible renal arteries. Additionally, incorrect initial deployment of the graft will increase procedure times, resulting in increased anesthesia and contrast usage. Bilateral renal artery stenting before FEVAR allows for a targeted docking of the graft, virtually eliminating any radiographic uncertainty and exposure to excess intraprocedural troubleshooting. Traditionally, renal stenting for therapeutic purposes leaves an approximate stent extension of approximately 2 mm into the aorta in an attempt to preserve long-term patency for targeting purposes; the stent should be deployed flush with the orifice of the renal artery to ensure it does not have contact with the incoming aortic graft, thus avoiding renal stent proximal end crush damage. Preoperative stenting before AAA repair does add another procedure and its expectant cost to the patient. Without accurate graft deployment, there is also an increased likelihood of the previously mentioned procedural complications. Renal artery occlusion may even result in long-term dialysis, a financial burden to the patient. Endoleak may require additional balloon angiography and graft remodeling to achieve an adequate seal [20]. Failed revision attempts may require embolization or open surgical repair [20]. Targeted graft placement on the first attempt and avoidance of these complications outweighs the cost difference of preoperative stent placement.

Initial primary renal stenting allowed our team to efficiently complete fenestrated endovascular aortic repairs without revision or postoperative complications. This article's example case would not have been possible without the renal stent target in place. At our institution, we preoperatively stent the renal arteries for targeting purposes before our FEVAR cases. We believe the widespread use of this technique for all fenestrated graft placements will decrease the number of perioperative complications and increase the percentage of positive patient outcomes and technical success.

## Conclusions

Fenestrated grafts are the forefront medical device technology for the repair of abdominal aortic aneurysms. Endovascular deployment of these grafts present challenges due to hostile patient anatomy leading to prolonged procedure times, endoleak, and possible occlusion of the renal vasculature. Stenting the patients’ renal arteries for targeting purposes before the endograft procedure allows for the device’s timely and precise deployment. In cases where deployment position is not satisfactory, prior stenting will aid in targeted repositioning maneuvers. Future studies will demonstrate this technique's ability to improve patient outcomes and technical success while minimizing postoperative complications.
